# Characterization and selection of functional yeast strains during sourdough fermentation of different cereal wholegrain flours

**DOI:** 10.1038/s41598-020-69774-6

**Published:** 2020-07-30

**Authors:** Michela Palla, Massimo Blandino, Arianna Grassi, Debora Giordano, Cristina Sgherri, Mike Frank Quartacci, Amedeo Reyneri, Monica Agnolucci, Manuela Giovannetti

**Affiliations:** 1 Department of Agriculture, Food and Environment, University of Pisa, Via del Borghetto 80, 56124 Pisa, Italy; 2 Department of Agricultural, Forest and Food Sciences (DISAFA), Università Degli Studi Di Torino, Largo Braccini 2, 10095 Grugliasco, TO Italy; 3 Interdepartmental Research Centre “Nutraceuticals and Food for Health”, University of Pisa, Pisa, Italy

**Keywords:** Microbiology, Applied microbiology, Biochemistry

## Abstract

The increasing demand for healthy baked goods boosted studies on sourdough microbiota with beneficial metabolic traits, to be used as potential functional starters. Here, 139 yeasts isolated from cereal-based fermented foods were in vitro characterized for their phytase and antioxidant activities. The molecular characterization at strain level of the best 39 performing isolates showed that they did not derive from cross contamination by baker’s yeast. Afterwards, the 39 isolates were in vivo analyzed for their leavening ability, phytase activity and polyphenols content using five different wholegrain flours, obtained from conventional and pigmented common wheat, emmer and hull-less barley. Combining these findings, through multivariate permutation analysis, we identified the 2 best performing strains, which resulted diverse for each flour. Doughs singly inoculated with the selected strains were further analyzed for their antioxidant capacity, phenolic acids, xanthophylls and anthocyanins content. All the selected yeasts significantly increased the total antioxidant activity, the soluble, free and conjugated, forms of phenolic acids and anthocyanins of fermented doughs. This study revealed the importance of a specific selection of yeast strains for wholegrain flours obtained from different cereals or cultivars, in order to enhance the pro-technological, nutritional and nutraceutical traits of fermented doughs.

## Introduction

Fermentation is one of the oldest methods used by humans since ancient times for preserving foods and improving their organoleptic properties. More than 5,000 fermented foods and beverages are produced all over the world, from wine, beer and vinegar to cheese, yogurt, sourdough bread, olives, sausages, kimchi and miso^1^. Fermentation consists of the biochemical modification of raw materials, promoted by a complex and stable consortium of microorganisms, which mainly transform sugars into simple acids, alcohols and carbon dioxide, improving flavor, texture and aroma, and prolonging the shelf-life of the fermented products^2^. During fermentation, a wide range of secondary metabolites, including vitamins, polyols, antioxidants and bioactive compounds are also produced by microbial communities, enhancing the nutritional and nutraceutical values of the final products^3^.

Among fermented foods, sourdough bread is one of the most important baked goods derived from cereal fermentation^4^. The estimated annual *pro capita* intake of bread in European countries is reported to range from 46 (Sweden, Great Britain, Finland and Austria) to 100 kg (Greece, Portugal, Spain and Italy)^5^, and about 30 to 50% of bread is manufactured using sourdough^6^. Sourdough is a mixture of cereal flour and water, fermented by a complex biological ecosystem, consisting of lactic acid bacteria (LAB) and yeasts which interact with each other, often establishing stable associations and contributing to the beneficial properties of sourdough breads^7^. Each sourdough harbors different LAB and yeast communities, whose diversity depends on the type of flour and water, processing environments and various process parameters such as temperature, time of fermentation, number of refreshments and so on. *Saccharomyces cerevisiae* is the yeast species most frequently retrieved, followed by *Kazachstania humilis* and *Wickerhamomyces anomalus*^8^, while among LAB, *Lactobacillus sanfranciscensis* represents the dominant species^9^. Different species and strains may have different metabolic patterns, conferring specific properties to each sourdough bread. For example, some strains synthesize essential amino acids or vitamins, i.e. thiamine, vitamin E and folates, others produce prebiotic exopolysaccharides (EPS) and bioactive compounds such as polyphenols, organic acids, peptides and amino acid derivatives, i.e. γ-amino butyric acid (GABA)^10^. Moreover, microbial strains may produce enzymes, i.e. proteases, phytases or lipases, and degrade anti-nutritional factors such as raffinose and phytic acid which chelates iron, calcium, magnesium and zinc, thus depleting their bioavailability^10^.

In recent years, the increasing demand for healthy foods boosted studies on sourdough microbiota with beneficial metabolic features to be used as potential functional starters for the production of baked goods. Most works focused on the functional and nutritional features of sourdough as the consequence of LAB metabolism^10, 11^, though an increasing number of studies highlighted the important role of yeast metabolism on such beneficial traits^8, 12^. Indeed, several yeast strains are able to produce different vitamins^13, 14^ and antioxidant compounds^15, 16^, and to degrade antinutritional substances such as phytates^17, 18^. Recently, Palla et al*.*^19^, investigating the pro-technological, nutritional and functional traits of 78 Tuscan sourdough yeasts, detected different *S. cerevisiae* strains showing phytase activity and antioxidant properties.

Wholegrain flour has a high content of bioactive compounds, in particular phenolic acids, the main responsible of the antioxidant activity of cereal grains, flours and derived baked products^20^. Moreover, anthocyanins and carotenoids, phytochemicals with a strong antioxidant activity, are also attracting an increased interest among wholegrain products derived from different cereals such as rice^21^, maize^22^ and, more recently, wheat^23, 24^. These bioactive compounds are generally found in conventional red-grained common wheat (*Triticum aestivum* L. spp. *aestivum*) varieties; however, purple or blue-pigmented grain cultivars show a high content of anthocyanins in both pericarp and aleurone layers, respectively^23^. Carotenoids are responsible for the yellow colour of the endosperm and flour of durum wheat (*Triticum turgidum* spp. *durum* (Desf.) Husn*.*) and other diploid and tetraploid *Triticum* species such as einkorn (*T. monococcum* L. spp. *monococcum*) and emmer (*T. turgidum* spp*. dicoccum* (Schrank) Thell). Carotenoids have been detected in high concentrations also in yellow-grained cultivars of common wheat^23^. Barley, in particular hull-less barley (*Hordeum vulgare* L. var. *nudum* Hook), is an excellent source of soluble and insoluble fibers and shows a high antioxidant activity, its flour representing a functional ingredient for bakery products^25^.

The overall objective of this work was the selection of yeast strains with interesting functional and nutritional traits to be used as functional starters for the production of healthy breads. With this aim, 139 yeast strains isolated from cereal-based fermented foods and drinks, were characterized and selected for their pro-technological, functional and molecular traits by in vitro and in vivo screening. Five different wholegrain flours were used: a conventional red-grained wheat variety, a yellow-grained wheat variety rich in carotenoids, a blue-grained wheat variety rich in anthocyanins, a winter emmer variety and a hull-less spring barley variety.

## Results

### Functional characterization of flours

In order to evaluate the functional properties of the five wholegrain flours, their antioxidant capacity, phenolic acid, xanthophyll and anthocyanin contents were analyzed (Supplementary Table S1 online). Hull-less barley flour showed the highest antioxidant activity, considering both *ferric reducing antioxidant power (*FRAP) and ABTS *[2,2′-Azino-bis (3-ethylbenzthiazoline-6-sulfonic acid)]* methods, and a cell wall-bound phenolic acids (CWBPA) content significantly higher compared to the other cultivars, except for the blue-grained wheat. The content of soluble phenolic acids (SPAs) was the lowest one due to a significantly lower concentration of sinapic acid if compared to the other cereals. On the other hand, emmer flour had overall the lowest FRAP antioxidant activity and CWBPA content, and a significantly lower concentration of lutein and zeaxanthin compared to the yellow-grain common wheat flour. Furthermore, emmer flour had a SPA content, in particularly sinapic acid, significantly higher than hull-less barley and yellow-grained common wheat flours. The blue-grained cultivar Skorpion showed a significantly higher concentration of SPAs and CWBPAs than the other common wheat flours. Although the blue-grained and yellow-grained wheat cultivars showed a higher content of anthocyanins and xanthophyll, respectively, the antioxidant activity measured with both methods was not significantly different among the common wheat cultivars.

### In vitro functional characterization of yeast isolates

Phytase activity, evaluated on 139 yeast isolates, was detected in 77% of isolates, 40% of which showed a *halo zone* higher than 2 mm (Supplementary Table S2 online). The results of the antioxidant activity showed a high variability among the 139 yeast isolates: 75% of them exhibited a radical scavenging capacity, ranging from 65.11 ± 14.43 to 0.34 ± 0.34 nmol TEAC/mL (Trolox equivalents of antioxidant capacity).

### Molecular characterization of selected yeast isolates

According to in vitro preliminary results, 55 yeast isolates showing the highest values for phytase and/or antioxidant activities were selected. Among them, 31 isolates had been previously molecularly characterized^19^. The other 24 isolates, still of unknown identity, were analyzed by the amplification of the D1/D2 domains of the 26S rRNA gene and sequencing. An amplicon of about 600 bp was obtained for each isolate, as expected. The amplicons were sequenced and affiliated to fungal species using BLAST and phylogenetic trees analyses (Table 1). Sequences were affiliated with *Candida parapsilosis* (46%), *S. cerevisiae* (17%)*, Pichia fermentans* (8%)*, Prillingera fragicola* (8%)*, Candida sake* (4%)*, Candida inconspicua* (4%)*, Torulaspora quercuum* (4%), *Rhodotorula mucilaginosa* (4%) and *Cutaneotrichosporon curvatus* (4%).

**Table 1:** Phylogenetic identification of 24 yeast isolates showing optimal functional traits in vitro.

Isolate	GeneBank Accession N°	Identification	Identity (%)	Most closely related GeneBank sequences
BL2	MT269548	*Prillingera fragicola* CBS:8898	99%	NG_058427
BL3	MT269550	*Candida sake* CBS:5690	100%	KY106734
BL4	MT269549	*Prillingera fragicola* CBS:8898	99%	NG_058427
BL5	MT269551	*Saccharomyces cerevisiae* A60	100%	KM589486
BL7	MT269552	*Saccharomyces cerevisiae* CBS:2962	100%	KY109317
BL10	MT269555	*Candida inconspicua* CBS:2833	100%	KY106513
BL11	MT269556	*Pichia fermentans* CBS:1876	99.66%	KY108811
BL12	MT269558	*Torulaspora quercuum* CBS:11403	100%	NG_058415
BL14	MT269557	*Pichia fermentans* CBS:1876	100%	KY108811
BL16	MT269559	*Rhodotorula mucilaginosa* CBS:482	99.83%	KY109140
BL19	MT269560	*Cutaneotrichosporon curvatum* CBS:570	100%	AJ555468
GTS3Y	MT269561	*Candida parapsilosis* CBS:7156	99.49%	KY106673
GTS6Y	MT269562	*Candida parapsilosis* CBS:7156	100%	KY106673
GTS9Y	MT269563	*Candida parapsilosis* CBS:7156	100%	KY106673
GTS11Y	MT269564	*Candida parapsilosis* CBS:7156	100%	KY106673
GTS12Y	MT269565	*Candida parapsilosis* CBS:7156	100%	KY106673
GTS18Y	MT269566	*Candida parapsilosis* CBS:7156	99.67%	KY106673
GTS20Y	MT269567	*Candida parapsilosis* CBS:7156	100%	KY106673
GTSXY	MT269553	*Saccharomyces cerevisiae* CBS:2962	99.67%	KY109317
GTW10Y	MT269568	*Candida parapsilosis* CBS:7156	100%	KY106673
GTW13Y	MT269569	*Candida parapsilosis* CBS:7156	100%	KY106673
GTW14Y	MT269570	*Candida parapsilosis* CBS:7156	100%	KY106673
GTW15Y	MT269571	*Candida parapsilosis* CBS:7156	100%	KY106673
W9DY	MT269554	*Saccharomyces cerevisiae* CBS:2962	100%	KY109317

On the basis of the molecular identification, eight out of the 24 isolates were selected as belonging to species commonly found in sourdoughs (*S. cerevisiae, P. fermentans, C. sake* and *T. quercuum*) and further characterized at the strain level by inter-delta regions analysis, together with the 31 already characterized. The dendrogram, obtained comparing the inter-delta profiles of such isolates, along with the profiles of the two commercial baker’s yeasts, showed a high intraspecific diversity, discriminating 20 *S. cerevisiae* biotypes (Supplementary Fig. S1 online). It is interesting to note that none of our isolates shared the profile with the two commercial baker’s yeast strains.

### Leavening ability of selected yeast strains

**Figure 1:**
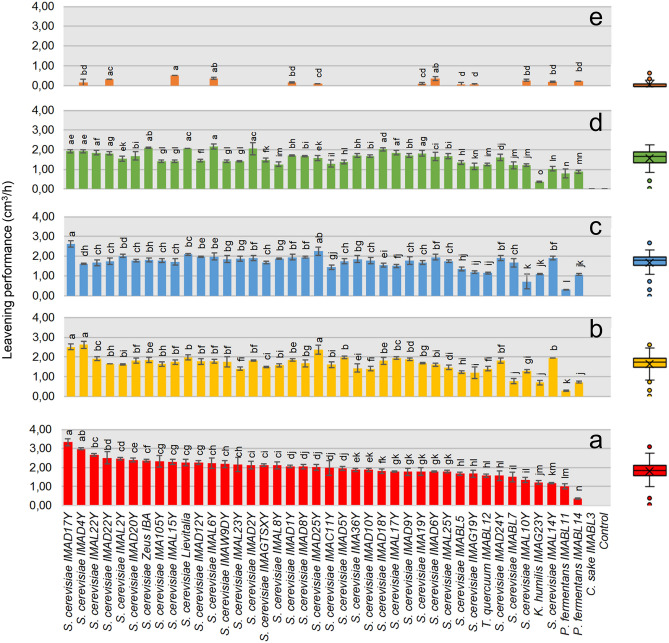
Leavening ability (cm^3^/h) of (**a**) conventional red-grained wheat variety *T. aestivum* L. cv. Aubusson, (**b**) yellow-grained wheat variety *T. aestivum* L. cv. Bona Vita, (**c**) blue-grained wheat variety *T. aestivum* L. cv. Skorpion, (**d**) winter emmer variety *T. turgidum* subsp. *dicoccum* var. *Schrank*, Giovanni Paolo and (**e**) hull-less spring barley variety *H. vulgare* L. var. *nudum* Hook, Rondo fermented doughs individually inoculated with selected yeast strains. Control, Lievitalia and Zeus IBA represent doughs not inoculated and inoculated with commercial baker’s yeasts, respectively. Bars of standard deviations are also represented. Data followed by the same letter do not differ (welch = (**a**) 41.04, *p* < 0.001; (**b**) 106.14, *p* < 0.001; (**c**) 59.45, *p* < 0.001; (**d**) 64.21, *p* < 0.001; (**e**) 207.46, *p* < 0.001) Boxplot with mean ( ×) and median (black line) are shown. White spots correspond to minimum and maximum values.

The 39 molecularly characterized isolates were further analyzed for their leavening ability by in vivo screening using the five wholegrain flours. Results are reported in Fig. 1. All strains inoculated in wheat and emmer flours, except *C. sake* IMA BL3, showed leavening ability ranging from 3.34 ± 0.18 cm^3^/h in the case of the conventional red-grained wheat cv. Aubusson flour fermented by *S. cerevisiae* IMA D17Y to 0.27 ± 0.03 cm^3^/h for the yellow-grained common wheat cv. Bona Vita flour fermented by *P. fermentans* IMA BL11 (Figs. 1a–d). Only 33% of our yeasts inoculated in barley flour showed such a capacity (≤ 0.51 cm^3^/h), while the commercial baker’s yeasts did not show any leavening ability (Fig. 1e). Overall, the highest leavening performances were shown by yeasts belonging to the *S. cerevisiae* species. Moreover, 72, 82 and 72% of the strains inoculated into the common wheat Aubusson, Skorpion and Bona Vita, respectively, showed the same or a significantly higher leavening performance, compared to the commonly used commercial baker’s yeasts. It is interesting to note that *S. cerevisiae* IMA D17Y, D4Y and L6Y showed the highest values of leavening ability in almost all the doughs.

The average pH values at the end of the fermentation were about 5.89 ± 0.01 in common wheat cultivars and in emmer fermented doughs, while doughs made using hull-less barley flours showed pH values generally lower (mean 5.56 ± 0.01).

### In vivo functional properties of selected yeast strains

For each flour, the 10 strains with the highest leavening ability were selected and analyzed for their phytase activity and polyphenol content by in vivo screening.

**Figure 2:**
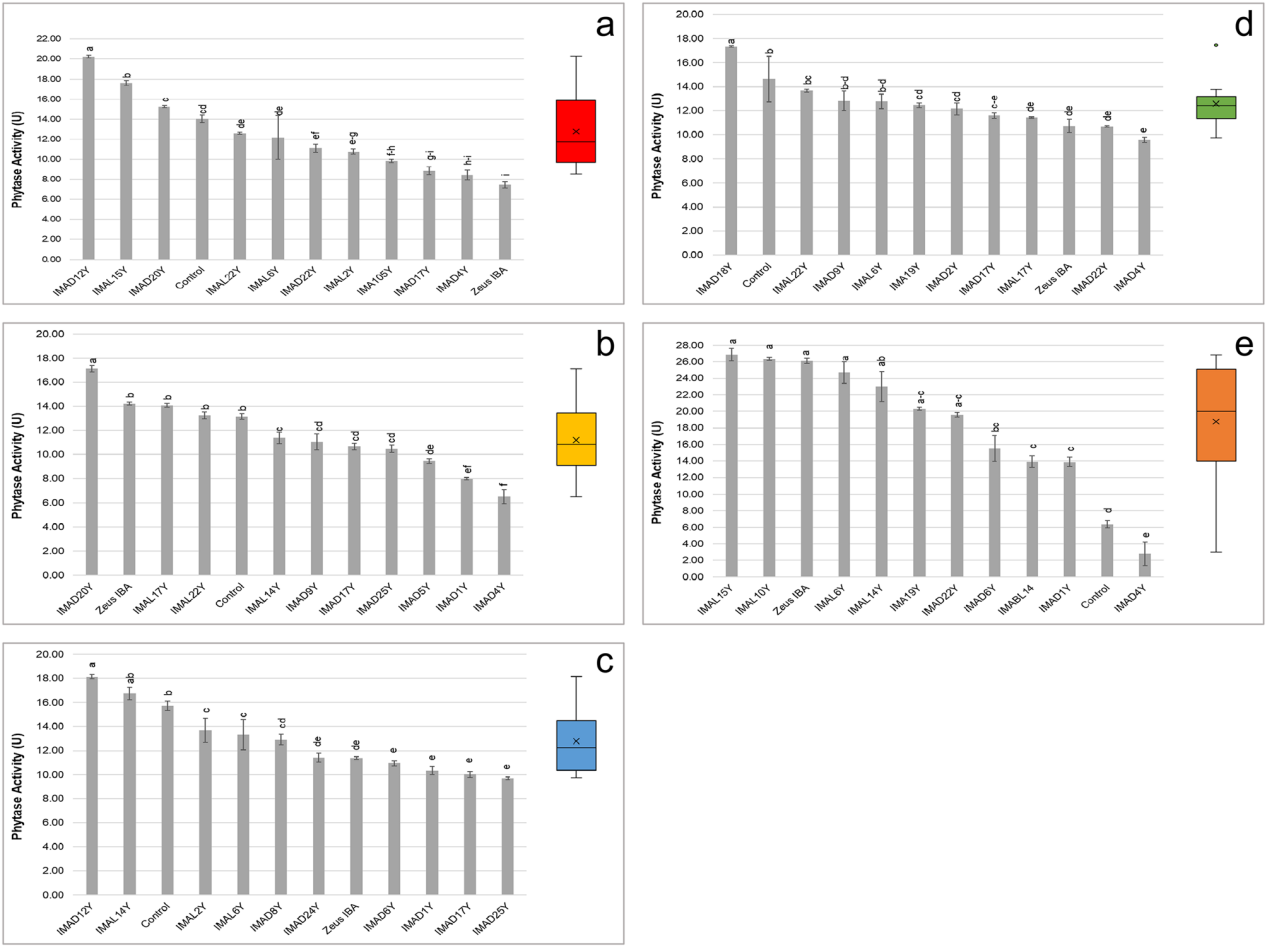
Phytase activity of (**a**) conventional red-grained wheat variety *T. aestivum* L. cv. Aubusson, (**b**) yellow-grained wheat variety *T. aestivum* L. cv. Bona Vita, (**c**) blue-grained wheat variety *T. aestivum* L. cv. Skorpion, (**d**) winter emmer variety *T. turgidum* subsp. *dicoccum* var. *Schrank*, Giovanni Paolo and (**e**) hull-less spring barley variety *H. vulgare* L. var. *nudum* Hook, Rondo fermented doughs individually inoculated with selected yeast strains. Control and Zeus IBA represent doughs not inoculated and inoculated with commercial baker’s yeasts, respectively. One unit (U) of activity was defined as the amount of enzyme required to liberate 1 μmol/min of p-nitrophenol under the assay conditions. Bars of standard deviations are also represented. Data followed by the same letter do not differ (welch = (**a**) 246.16, *p* < 0.001; (**c**) 123.24, *p* < 0.001; (**d**) 513.09, *p* < 0.001; (**e**) 78.54, *p* < 0.001; (**b**) F = 70.73, *p* < 0.001) Boxplot with mean ( ×) and median (black line) are shown. White spots correspond to minimum and maximum values.

Phytase activity was determined on the water-salt soluble extract of fermented doughs by monitoring the rate of hydrolysis of *p*-nitrophenyl phosphate (*p*-NPP). Results showed a high variability, ranging from 26.9 ± 0.7 to 2.8 ± 1.4 U among the doughs started with the different strains (Fig. 2), the highest values being reached in doughs obtained with the hull-less barley cv. Rondo flour. Moreover, 80 and 40% of strains inoculated in the red-grained cv. Aubusson (Fig. 2a) and blue-grained cv. Skorpion (Fig. 2c) flours, respectively, exhibited phytase activity values significantly higher compared to the commercial baker’s yeast Zeus IBA.

**Figure 3:**
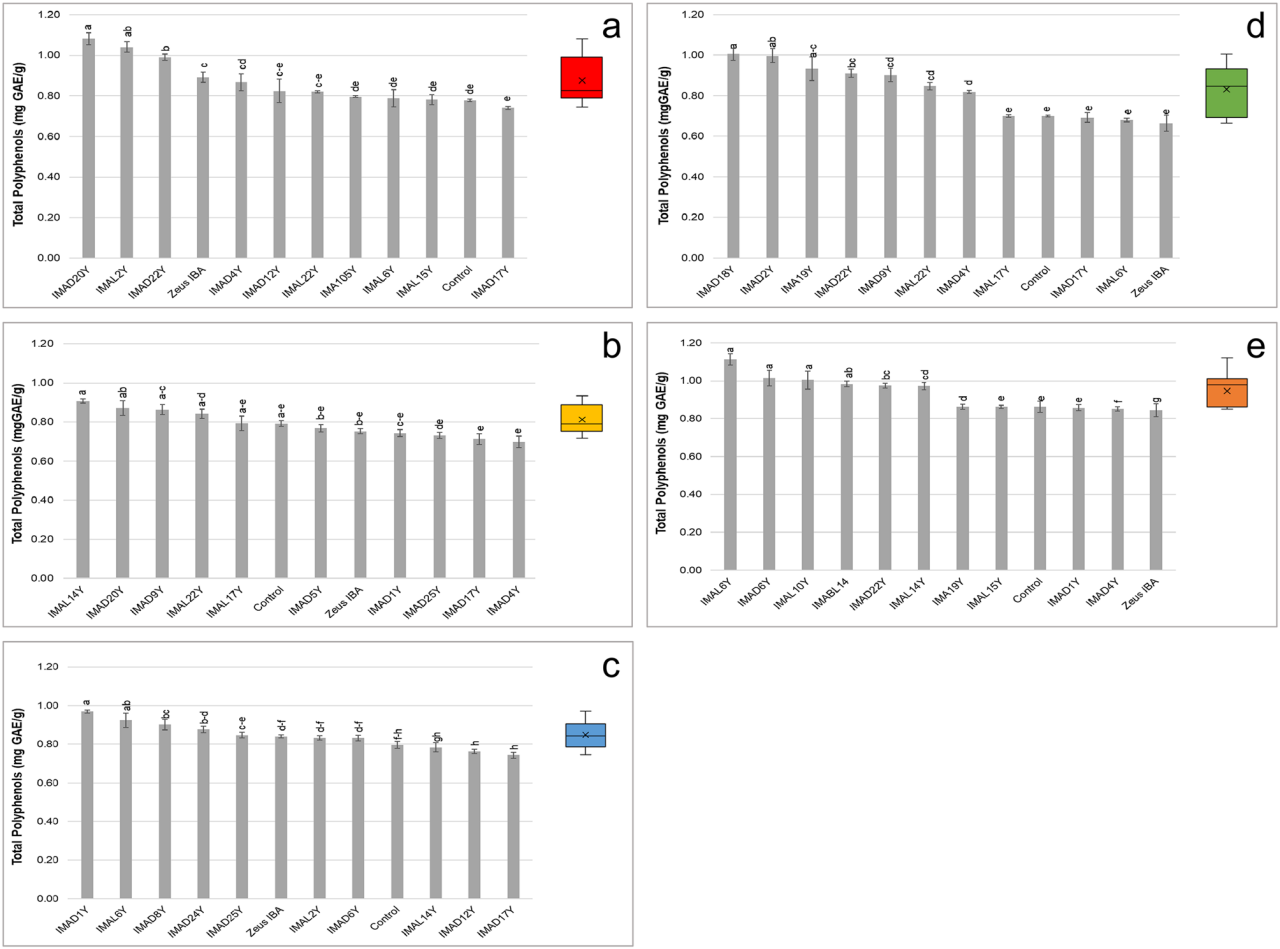
Total phenols (mg GAE/g) of (**a**) conventional red-grained wheat variety *T. aestivum* L. cv. Aubusson, (**b**) yellow-grained wheat variety *T. aestivum* L. cv. Bona Vita, (**c**) blue-grained wheat variety *T. aestivum* L. cv. Skorpion, (**d**) winter emmer variety *T. turgidum* subsp. *dicoccum* var. *Schrank*, Giovanni Paolo and (**e**) hull-less spring barley variety *H. vulgare* L. var. *nudum* Hook, Rondo fermented doughs individually inoculated with selected yeast strains. Control and Zeus IBA represent doughs not inoculated and inoculated with commercial baker’s yeasts, respectively. Bars of standard deviations are also represented. Data followed by the same letter do not differ (welch = (**a**) 22.49, *p* < 0.001; (**c**) 21.82, *p* < 0.001; (**d**) 27.12, *p* < 0.001; (**e**) 144.63, *p* < 0.001; (**b**) F = 7.96, *p* < 0.001) Boxplot with mean ( ×) and median (black line) are shown.

The analysis of the total phenol content was carried out on the methanolic extracts of fermented doughs by monitoring the reaction between phenols with the Folin-Ciocalteu reagent. Results showed that almost all the strains led to an increase in the total phenol content, ranging from 1.08 ± 0.03 to 0.68 ± 0.01 mg GAE/g (GAE: gallic acid equivalents) (Fig. 3). It is interesting to note that three strains for the conventional red-grained common wheat (Fig. 3a), three for the blue-grained cv. Skorpion (Fig. 3c), seven for emmer (Fig. 3d) and all strains for barley (Fig. 3e) sourdoughs showed polyphenol contents significantly higher than the commercial baker’s yeast.

**Figure 4:**
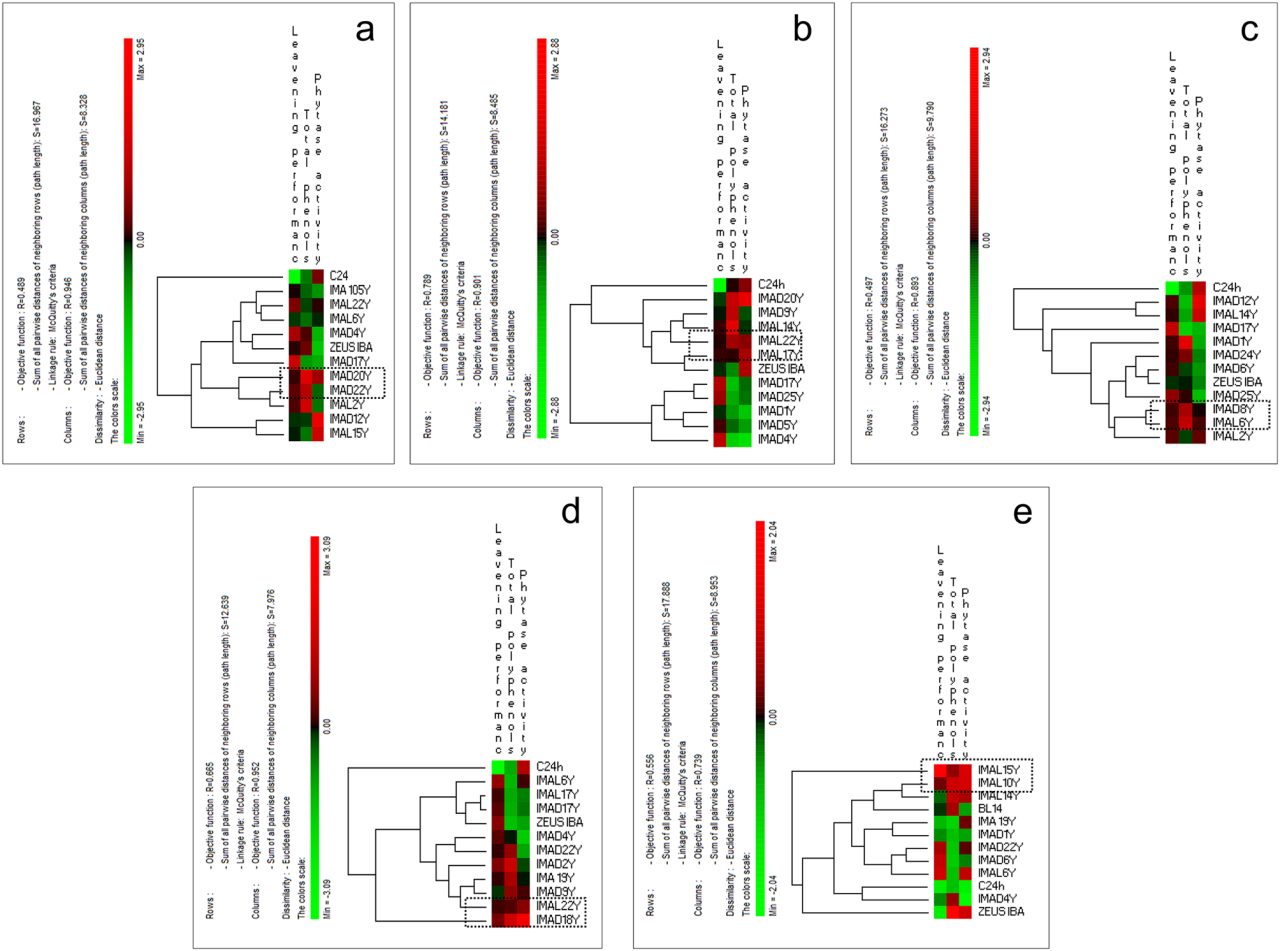
Leavening ability (cm^3^/h), phytase activity (U) and total phenols (mg GAE/g) (**a**) conventional red-grained wheat variety *T. aestivum* L. cv. Aubusson, (**b**) yellow-grained wheat variety *T. aestivum* L. cv. Bona Vita, (**c**) blue-grained wheat variety *T. aestivum* L. cv. Skorpion, (**d**) winter emmer variety *T. turgidum* subsp. *dicoccum* var. *Schrank*, Giovanni Paolo and (**e**) hull-less spring barley variety *H. vulgare* L. var. *nudum* Hook, Rondo fermented doughs individually inoculated with selected yeast strains. Euclidean distance and McQuitty’s criterion (weighted pair group method with averages) were used for clustering. Colours correspond to normalized mean data levels from low (green) to high (red). The colour scale, in terms of units of standard deviation, is shown on the left side. Strains marked by the dashed box are the best performing strains.

Combining the leavening ability, phytase activity and phenol content values through multivariate permutation analysis, the two best performing strains for each flour were identified (Fig. 4). In particular, *S. cerevisiae* IMA D20Y and D22Y (Fig. 4a), *S. cerevisiae* IMA L22Y and L17Y (Fig. 4b), *S. cerevisiae* IMA D8Y and L6Y (Fig. 4c), *S. cerevisiae* IMA L22Y and D18Y (Fig. 4d), *S. cerevisiae* IMA L15Y and L10Y (Fig. 4e) resulted the best performing yeasts when inoculated in the red-grained wheat cv. Aubusson, yellow-grained wheat cv. Bona Vita, blue-grained wheat cv. Skorpion, emmer cv. Giovanni Paolo and barley cv. Rondo flours, respectively. Interestingly, the best performing yeast strains were different for each wholegrain flour.

Doughs singly inoculated with such yeast strains were further analyzed for their antioxidant capacity and phenolic acid, xanthophyll and anthocyanin contents. Fermentation with yeasts resulted in a significant increase in CWBPA content only for the conventional red-grained common wheat, although a significant effect on single phenolic acids was found in all the considered cereals (Table 2). The CWBPAs that increased after yeast fermentation were hydroxybenzoic and syringic acids (in all common wheat cv. and emmer), vanillic acid (in red-grained common wheat, emmer and barley), *p*-coumaric acid (only in emmer) and sinapic acid (only in blue-grained common wheat). Ferulic acid, the main CWBPAs detected in all cereals, showed significant changes in doughs obtained with the red-grained cv. Aubusson flour.

**Table 2:** Cell wall-bound phenolic acids (CWBPAs) of five different cereal fermented doughs individually inoculated with selected yeast strains.

Cereal flour	Yeast strains^a^	CWBPAs^b^ (mg/kg)	Hydroxybenzoic acid (mg/kg)	Vanillic acid (mg/kg)	Caffeic acid (mg/kg)	Syringic acid (mg/kg)	*p*-coumaric acid (mg/kg)	Ferulic acid (mg/kg)	Sinapic acid (mg/kg)
Red-grained Common wheat (cv. Aubusson)	Control	826c	3.00b	5.57b	7.52a	2.76b	22.8a	720c	64.7a
	ZEUS IBA	957a	3.55a	6.57a	7.72a	3.47a	25.5a	837a	72.5a
	D20Y	917ab	3.66a	6.10ab	7.58a	3.13ab	23.8a	810ab	62.8a
	D22Y	886b	3.34ab	5.96b	7.83a	2.98b	24.3a	774b	66.8a
	SEM	13	0.12	0.15	0.35	0.11	0.7	12	2.1
	P(F)	0.001	0.017	0.01	0.914	0.012	0.147	0.001	0.051
Yellow-grained common wheat (cv. Bona vita)	Control	858a	3.94c	7.16a	6.41a	6.22b	30.4a	759a	45.0a
	ZEUS IBA	837a	4.22b	7.11a	6.88a	6.7a	28.4a	727a	55.9a
	L17Y	896a	4.56a	7.56a	7.02a	6.99a	30.0a	784a	55.8a
	L22Y	868a	4.4ab	7.44a	7.34a	6.9a	29.5a	761a	51.9a
	SEM	22	0.05	0.15	0.23	0.09	0.7	20	3.1
	P(F)	0.35	< 0.001	0.186	0.115	< 0.001	0.27	0.33	0.11
Blue-grained common wheat (cv. Skorpion)	Control	866a	5.29b	7.87a	6.42a	6.38b	39.0a	768a	33.1b
	ZEUS IBA	890a	6.30a	8.53a	6.87a	7.8a	40.4a	782a	37.5a
	D8Y	907a	5.80ab	8.10a	6.86a	7.57a	39.8a	800a	39.4a
	L6Y	924a	6.42a	8.54a	6.56a	7.64a	42.8a	814a	37.7a
	SEM	19	0.2	0.27	0.21	0.19	1.2	18	0.9
	P(F)	0.236	0.015	0.292	0.406	0.002	0.198	0.35	0.006
Emmer (cv. Giovanni Paolo)	Control	588a	2.33b	4.42b	4.48a	2.48b	17.9b	524a	33.0a
	ZEUS IBA	670a	3.35a	5.40a	5.58a	3.02a	22a	594a	36.6a
	D18Y	649a	3.06a	5.22a	5.12a	2.89a	20.3ab	576a	36.7a
	L22Y	683a	3.37a	5.41a	5.58a	3.06a	22.8a	605a	37.6a
	SEM	21	0.1	0.19	0.28	0.08	1.0	19	2.1
	P(F)	0.051	< 0.001	0.019	0.076	0.004	0.031	0.066	0.447
Hull-less barley (cv. Rondo)	Control	985a	2.86a	7.22b	37.3a	2.09a	32.9a	884a	18.8a
	ZEUS IBA	1039a	3.58a	9.25a	38.3a	2.13a	33.7a	932a	19.8a
	L10Y	994a	3.08a	8.61a	36.6a	1.98a	33.3a	890a	21.1a
	L15Y	1001a	3.33a	8.92a	38.1a	2.22a	32.6a	894a	22.0a
	SEM	21	0.18	0.22	1.4	0.17	0.9	19	1.9
	P(F)	0.372	0.099	0.001	0.794	0.81	0.827	0.323	0.654

Data are expressed on a dw basis. For each cereal type, means followed by different letters are significantly different, according to the Tukey’s HSD test (the ANOVA level of significance is shown in the table).

*SEM* standard error of the mean.

^a^Control, dough obtained from water and whole grain flour mixture, without yeast addition; ZEUS IBA, *S. cerevisiae* commercial baker’s yeasts.

^b^Sum of the CWBPAs determined by means of the RP-HPLC/DAD.

The strongest effect of yeast fermentation was detected on SPAs dough content: in all the considered wholegrain flours and for all the compared yeasts, fermentation resulted in significant increases in SPAs, compared with the relevant controls (Table 3). Significant effects were reported for all the compared cereal flours for sinapic, hydroxybenzoic and vanillic acids. Interestingly, significantly higher increases in SPAs were detected for emmer dough when inoculated with the L22Y strain.

**Table 3:** Soluble (free and conjugated forms) phenolic acids (SPAs) of five different cereal fermented doughs individually inoculated with selected yeast strains.

Cereal flour	Yeast strains^a^	SPAs^b^(mg/kg)	Hydroxybenzoic acid (mg/kg)	Vanillic acid (mg/kg)	Syringic acid (mg/kg)	p-coumaric acid (mg/kg)	Ferulic acid (mg/kg)	Sinapic acid (mg/kg)
Red-grained common wheat (cv. Aubusson)	Control	148b	5.71c	8.55b	3.82b	3.24b	89.5a	37.1c
	ZEUS IBA	161a	7.89a	9.13a	4.82a	3.51a	91.7a	43.6a
	D20Y	157a	7.33b	9.07a	4.66a	3.30b	92.1a	40.8b
	D22Y	162a	7.60ab	9.08a	4.61a	3.42ab	95.7a	42.0ab
	SEM	2	0.14	0.13	0.07	0.05	1.3	0.5
	P(F)	0.004	< 0.001	0.033	< 0.001	0.019	0.053	< 0.001
Yellow-grained common wheat (cv. Bona vita)	Control	153b	7.46b	12.3b	9.04b	2.98a	82.2a	39.1b
	ZEUS IBA	162a	10.14a	13.6a	11.09a	3.18a	80.1a	44.4a
	L17Y	167a	10.30a	13.7a	11.73a	3.36a	83.3a	44.6a
	L22Y	167a	10.03a	13.8a	11.41a	3.29a	83.7a	44.8a
	SEM	2	0.11	0.2	0.25	0.12	1	0.6
	P(F)	0.002	< 0.001	0.001	< 0.001	0.22	0.11	< 0.001
BLUE-grained common wheat (cv. Skorpion)	Control	146b	6.89c	11.3b	9.56b	3.2b	80.4a	35.1b
	ZEUS IBA	162a	9.85ab	12.7a	12.5a	3.43ab	82.0a	41.2a
	D8Y	159a	9.54b	12.7a	12.2a	3.39ab	79.6a	41.2a
	L6Y	162a	9.96ab	12.9a	12.8a	3.59a	80.9a	41.4a
	SEM	2	0.05	0.16	0.25	0.07	0.8	0.8
	P(F)	0.002	< 0.001	< 0.001	< 0.001	0.023	0.266	0.001
Emmer (cv. Giovanni Paolo)	Control	112c	5.33b	7.76b	2.53a	3.33c	54.4c	38.5c
	ZEUS IBA	131b	7.09a	9.28a	2.94a	3.66b	63.4b	44.4ab
	D18Y	135ab	7.03a	9.44a	2.83a	4.20a	67.6ab	43.5b
	L22Y	141a	7.23a	9.27a	3.00a	3.97a	69.9a	48.1a
	SEM	2	0.08	0.17	0.12	0.07	1.2	1
	P(F)	< 0.001	< 0.001	< 0.001	0.096	< 0.001	< 0.001	0.001
Hull-less barley (cv. Rondo)	control	126c	3.00b	8.03b	1.53b	6.09ab	94.8b	12.1b
	ZEUS IBA	140a	4.00a	9.99a	1.94a	6.55a	103a	13.8a
	L10Y	130bc	3.92a	9.75a	1.88a	5.85bc	95b	13.7a
	L15Y	133b	3.98a	9.91a	1.94a	5.34c	98.5b	13.4a
	SEM	1	0.08	0.16	0.06	0.14	1	0.3
	P(F)	0.001	< 0.001	< 0.001	0.006	0.002	0.001	0.006

Data are expressed on a dw basis. For each cereal type, means followed by different letters are significantly different, according to the Tukey’s HSD test (the ANOVA level of significance is shown in the table).

*SEM* standard error of the mean.

^a^Control, dough obtained from water and whole grain flour mixture, without yeast addition; ZEUS IBA, *S. cerevisiae* commercial baker’s yeasts.

^b^Sum of the SPAs determined by means of the RP-HPLC/DAD.

Yeast fermentation did not affect lutein and zeaxanthin dough content, while it significantly increased total anthocyanin content (TAC) in the blue-grained common wheat flour (Table 4). No effect was reported for antioxidant capacity measured with the ABTS method. On the contrary, fermentation with some selected strains significantly increased antioxidant capacity measured with the FRAP method in all the wholegrain flours, with the exception of that obtained from the yellow-grain common wheat. It is interesting to note that barley flour fermented by the L10Y strain showed the highest content of antioxidant capacity. Moreover, L6Y and L22Y strains showed significantly higher antioxidant capacity compared to the controls when inoculated in blue-grained wheat cv. Skorpion and emmer flours.

**Table 4:** Total anthocyanin (TAC), xanthophyll (lutein and zeaxanthin) content and antioxidant capacity (AC) of five different cereal fermented doughs individually inoculated with selected yeast strains.

Cereal flour	Yeast strains^a^	TAC (mg Cy-3-glc/kg)	Lutein (mg/kg)	Zeaxanthin (mg/kg)	AC-FRAP (mmol TE/kg)	AC-ABTS (mmol TE/kg)
Red-grained common wheat (cv. Aubusson)	Control				11.5b	19.9a
	ZEUS IBA				12.6a	20.5a
	D20Y				12.5a	19.8a
	D22Y				13.1a	20.6a
	SEM				0.2	0.6
	P(F)				0.013	0.713
Yellow-grained common wheat (cv. Bona vita)	Control		1.13a	0.168a	12.2a	21.2a
	ZEUS IBA		1.21a	0.158a	11.8a	20.7a
	L17Y		1.13a	0.162a	12.7a	21.6a
	L22Y		1.14a	0.157a	11.8a	21.0a
	SEM		0.03	0.005	0.4	0.5
	P(F)		0.18	0.51	0.28	0.62
Blue-grained common wheat (cv. Skorpion)	Control	17.7c			10.9b	20.2a
	ZEUS IBA	20.4a			11.9ab	21.3a
	D8Y	19.3b			11.7ab	21.8a
	L6Y	20.3a			12.4a	21.3a
	SEM	0.1			0.3	0.6
	P(F)	< 0.001			0.031	0.318
Emmer (cv. Giovanni Paolo)	Control		1.05a	0.153a	6.8b	20.3a
	ZEUS IBA		1.04a	0.142a	7.9a	19.9a
	D18Y		1.07a	0.153a	7.4ab	20.8a
	L22Y		1.07a	0.141a	8.1a	19.6a
	SEM		0.03	0.004	0.2	0.4
	P(F)		0.794	0.115	0.006	0.232
Hull-less barley (cv. Rondo)	Control				25.1b	44.9a
	ZEUS IBA				25.3b	40.9a
	L10Y				27.0a	45.6a
	L15Y				25.6ab	40.2a
	SEM				0.4	3.5
	P(F)				0.036	0.627

Data are expressed on a dw basis. For each cereal type, means followed by different letters are significantly different, according to the Tukey’s HSD test (the ANOVA level of significance is shown in the table).

*SEM* standard error of the mean.

^a^Control, dough obtained from water and whole grain flour mixture, without yeast addition; ZEUS IBA, *S. cerevisiae* commercial baker’s yeasts.

## Discussion

The increasing demand for healthy baked goods boosted studies on sourdough microbiota with beneficial metabolic traits, to be used as potential functional starters. The impact of fermentation technology on the functional properties has been disregarded so far, as most of the starter yeasts currently used are selected only for their peculiar leavening and other technological properties^26^. Here, we screened 139 yeast strains isolated from cereal-based fermented foods and drinks for their functional and nutritional traits, as affected by wholegrain flours obtained from five different cereals. Pro-technological, functional and molecular characterization allowed the selection of two yeast strains possessing, beyond a high leavening ability, the highest phytase and antioxidant activities, to be used as functional starters for the production of healthy baked goods.

The 139 yeasts were first in vitro characterized for their phytase and antioxidant activities. Phytic acid represents an abundant anti-nutritional factor in cereal flours which chelates minerals such as iron and zinc, depleting their bioavailability and uptake. Among our isolates, a high number of strains (77%) exhibited the ability to solubilize phytate, consistently with previous data showing such an activity in several sourdough yeast isolates, belonging mainly to *S. cerevisiae*^27, 28^ and in 13 out of 15 indigenous yeasts isolated from fermented foods^29^.

Over the past few years, the interest about the antioxidant properties of foods has strongly increased, as reactive oxygen species could be involved in many human diseases (e.g., cancer, diabetes, autoimmune conditions, etc.)^30^. Recently, yeasts have been shown to enhance bioactive components in fermented food products by the production of enzymes and metabolites that affect the antioxidant properties of baked goods^31^. Here, 75% of yeasts showed in vitro radical scavenging capability, with a high variability among them, consistently with data from Gazi et al*.*^32^ who, using the same method, found that 23 out of 25 yeast strains showed radical scavenging activity.

Based on the results of in vitro qualitative screening, 39 isolates were selected. Most of them belonged to *S. cerevisiae*, which is the most commonly species in backslopped sourdoughs^33^. Such isolates were molecularly characterized at the strain level by the inter-delta region analysis, which had been previously detected as the best discriminating technique to type sourdough *S. cerevisiae* isolates^19, 34^. Cluster analysis of the inter-delta profiles grouped all the *S. cerevisiae* isolates in a cluster showing a low similarity (44%) with the two commercial baker’s yeast strains, suggesting that our isolates could be autochthonous and did not derive from cross contamination by baker’s yeasts. Moreover, a high intraspecific diversity was detected, consistently with Osimani et al*.*^35^ and Pulvirenti et al*.*^36^, who used the same method to characterize, at the strain level, *S. cerevisiae* yeasts isolated from sourdoughs from the Italian regions Marche and Sicily, respectively. Similar results were also obtained by Palla et al*.*^19^, who observed a high polymorphism among 77 *S. cerevisiae* isolated from Tuscan sourdoughs.

The main role of yeasts in bread-making is gas production, which affects the volume of bread. Indeed, the most important trait, which is usually considered during yeast starter selection, is the ability to ferment sugars anaerobically with an adequate production of CO_2_, to ensure a uniform dough leavening^37^. *S. cerevisiae* is the species of choice in the baking industry due to its high fermentation capacity; indeed, it is characterized by a rapid consumption of sugars and a fast CO_2_ production, which are the most important attributes required to leaven the dough^38^. Here, almost all the selected isolates showed a high leavening ability, when inoculated in wheat and emmer flours. As expected, strains belonging to the *S. cerevisiae* species showed the best leavening performances compared to the other yeast genera and species. In particular, *S. cerevisiae* IMA D4Y, D17Y and D25Y showed a significant higher leavening performance than *S. cerevisiae* commercial baker’s yeast in doughs obtained using wheat flours, highlighting their efficiency as leavening agents. Similar results were found by Carbonetto et al*.*^39^, who reported that *K. humilis* strains never leavened dough as much as *S. cerevisiae* when singly inoculated in doughs. Moreover, our results showed that, contrary to *S. cerevisiae* commercial baker’s yeast, 33% of strains were able to increase the volume of doughs made using barley, which is a cereal with a lesser ability to form a gluten complex than wheat, thus decreasing dough gas retention^40^.

Besides carbon dioxide production, during fermentation yeasts produce ethanol and secondary metabolites which influence the quality of fermented products^41^. Yeasts affect the flavour of bread producing different aroma precursors such as esters, aldehydes and ketones; moreover, they increase the shelf-life producing various acids and glycerol^37^, and affect the functional and nutritional properties of bread producing enzymes and antioxidant compounds^31^. As the characterization of yeast strains with high leavening ability and able to produce functional enzymes and antioxidant compounds may lead to the selection of functional starters, ten yeasts for each flour showing the best performance in vitro were selected and further analyzed in vivo for their functional features (phytase activity and polyphenol content). Overall, all the yeast strains showed phytase activity, with a high variability among doughs started with the different strains. In the commercial baker’s yeast Zeus IBA phytase activity was strongly affected by the type of flour, highlighting the importance of selecting specific strains for each flour. Almost all the strains led to increases in sourdough total phenol contents, and at least one strain for each flour showed polyphenol contents significantly higher than those of the commercial baker’s yeast.

These findings are consistent with the recent literature, which reported that yeasts play an important role in increasing the polyphenol content of cereal products by releasing bound and conjugated phenolic acids to their free form after cell wall degradation process^42^. The analysis of the single phenolic acids carried out on wholegrain doughs showed that all the selected yeasts significantly increased the soluble, free and conjugated forms of phenolic acids. Among them, each single compound showed a significant increase, although at a different extent: within the main SPAs, sinapic acid showed a higher average increase (+ 15%) compared to ferulic acid (+ 6%), while among the minor compounds hydroxybenzoic and syringic acids increased by 35 and 24%, respectively. Our data are consistent with those reported by Skrajda-Brdak et al*.*^43^, who observed that free benzoic acids were preferentially accumulated in yeast fermented breads. In addition, SPA increases in fermented dough were higher in cereals characterized by a greater wholegrain flour SPA content, in particular for emmer. Since SPAs can be easier absorbed by the intestinal cells compared to bounded ones, their increase as a consequence of yeast fermentation may positively affect the amount of phenolics accessible in the upper gastrointestinal tract^44^.

SPA increases detected after dough fermentation did not correspond to a reduction in CWBPAs, which significantly increased in all the selected yeast strains compared to the control. This result was particularly evident for hydroxybenzoic and syringic acids, and for conventional red-grained common wheat cultivars. The CWBPAs increase could be ascribed to the production of phenolic compounds by yeasts, that could contribute to enhance their overall content in fermented doughs^45^.

In addition to phenolic acids, also anthocyanins increased in the fermented doughs, showing different amounts depending on the selected strain. Such data may have a functional significance as anthocyanins in pigmented wheat were recognized as health-enhancing substances due to their antioxidant, anti-inflammatory, anticancer and hypoglycemic activities^46^.

Several yeast species can produce different types of carotenoids such as astaxanthin, β-carotene, γ-carotene, torulene and torularhod^47^. In the present study, yeast fermentation did not seem to affect the content of lutein and zeaxanthin, the main carotenoids in wholegrain flour of yellow-grained wheat and emmer.

In accordance with the changes in the content of antioxidant compounds, a significant increase in total antioxidant activity was detected in fermented doughs, analyzed by means of the FRAP method. Such increases could be ascribed also to other compounds, i.e. small bioactive peptides produced by the action of proteolytic yeasts during dough fermentation^31^.

Combining all the results, for each of the five flours we selected two yeast strains with the highest leavening, phytase and antioxidant activities. It is interesting to note that different strains were detected for each flour, thus highlighting the importance of selecting specific strains for each cereal flour, each characterized by different biochemical and qualitative traits. Indeed, such strains had previously shown variable pro-technological and functional traits when individually used to ferment commercial soft and durum wheat flours^19^, confirming that the activity and production of enzymes during fermentation depend on the specificity of the substrate^31^.

In conclusion, this work highlighted the importance of a specific selection of yeast strains for wholegrain flours, obtained from different cereals or cultivars, in order to enhance the pro-technological, nutritional and nutraceutical traits of fermented doughs. With the aim of producing healthy baked goods, the overall strategy should entail the appropriate integration of wholegrain flour from cereals and cultivars with a higher content of bioactive compounds and antioxidants with selected yeast starter strains conferring the highest functional properties to fermented doughs.

## Materials and methods

### Grain cultivation and milling

In this study five different wholegrain flours were analyzed. Three varieties of an ordinary bread-making winter common wheat were used: a conventional red-grained variety (cv. Aubusson, Limagrain Italia SpA), a yellow-grained variety rich in carotenoids (cv. Bona Vita, Osivo a. s., Slovakia) and a blue-grained variety rich in anthocyanins (cv. Skorpion, Agricultural Research Institute Kromeriz, Ltd., Czech Republic). In addition, a winter emmer variety (cv. Giovanni Paolo, Apsovsementi s.p.a, Italy) and a hull-less spring barley variety (cv. Rondo, Società Italiana Sementi s.p.a, Italy) were also analyzed.

The five cereal cultivars were grown side by side on the same experimental field located in Carmagnola, Italy, (Piedmont; 44° 50′ N, 7° 40′ E; altitude 245 m) during the growing season 2016/2017. The plot size for each cultivar was 5 × 100 m (500 m^2^) and the ordinary crop technique of the growing area was applied. Wholegrain flour from each cultivar was obtained by means of a single-stream natural stone milling process (Molino Tomatis S.N.C, Niella Tanaro, Italy), without any sifting processes.

### Functional characterization of flours

The wholegrain flour from each cultivar was analyzed for SPA and CWBPA, xanthophylls, TAC and total antioxidant capacity. The extraction and quantification of soluble (free and conjugated) and cell wall-bound phenolic acids was performed according to the procedure proposed by Li et al*.*^48^ with some modifications as reported by Giordano et al.^49^. Briefly, one hundred and twenty-five milligrams of each sample were mixed in 1 mL of an 80:20 (v/v) ethanol:water solution and centrifuged at 10,600×*g* for 10 min, in order to remove the supernatants. A second extraction was carried out with 80:20 (v/v) ethanol:water solution. Supernatants were collected, hydrolyzed with 2 M NaOH (400 μL) for 2 h under continuous stirring at 4 °C, acidified with HCl, then SPAs were extracted with 500 μL of ethyl acetate.

The pellet remaining after the first part of the extraction was hydrolyzed for 4 h under continuous stirring at 4 °C by adding 2 M NaOH (400 μL). After acidification with HCl, the CWBPA were extracted with 800 μL of ethyl acetate and then centrifuged at 10,600×*g* for 2 min.

Phenolic extracts were filtered through a 0.2 μm filter and then analyzed by an Agilent 1200 Series (Agilent Technologies, Santa Clara, CA, USA) high-performance liquid chromatograph coupled to an Agilent 1200 Series diode array detector. Separations were carried out using a 150 × 4.6 mm, 5 μm particle size, Gemini RP-18 column (Phenomenex, Torrance, CA, USA).

The total anthocyanin content (TAC) was determined only for the stone-milled flour obtained from the blue-variety of common wheat (cv. Skorpion). Each sample (1 g) was extracted using 8 mL of ethanol acidified with 1 N HCl (85:15, v/v) for 30 min. The absorbance was measured after centrifugation at 20,800×*g* for 2 min at 540 nm as reported by Siebenhandl et al*.*^50^. TAC was expressed as mg cyanidin-3-*O*-glucoside (Cy-3-glc) equivalents/kg of sample (dw).

The extraction and quantification of xanthophylls was performed as previously reported by Giordano et al*.*^49^. The analysis was performed only for the stone-milled flours obtained from the yellow-grained variety of common wheat (cv. Bona Vita) and from the emmer variety (cv. Giovanni Paolo). *trans*-β-Apo-8′-carotenal was used as internal standard to ensure that losses due to the extraction method were accounted for.

The antioxidant capacity was determined by means of the ferric reducing antioxidant power (FRAP) and the ABTS [2,2′-Azino-bis (3-ethylbenzthiazoline-6-sulfonic acid)] assays adapted into QUENCHER method as described by Serpen et al*.*^51^.

### Microorganisms, media and growth conditions

The 139 yeasts used in this study were maintained in the International Microbial Archives (IMA) of the Microbiology Labs of the Department of Agriculture, Food and Environment (DAFE) of the University of Pisa (Supplementary Table S3 online) and originated as follows: 78 strains were previously isolated from wheat Tuscan sourdoughs^19^, 3 *S. cerevisiae* strains from PDO Tuscan bread sourdough^27^. The other 58 yeasts were isolated from different Italian sourdoughs (43) and from Boza, a cereal-based fermented drink (15), following the method described in Palla et al.^27^. Along with such isolates, two commercial baker’s yeasts—*S. cerevisiae* Zeus IBA (ZEUS IBA srl) and *S. cerevisiae* Lievitalia (Lesaffre Italia spa)—and four reference strains (Supplementary Table S3 online) were used. The isolates were grown in Yeast Extract Peptone Dextrose agar (YEPD: 1% yeast extract, 1% bacteriological peptone, 2% dextrose and 2% agar) at 28 °C for 48 h.

### In vitro functional characterization of yeast isolates

The 139 yeasts, along with the reference strains, were preliminary analyzed by in vitro screening for their functional traits, i.e. phytase and antioxidant activities. Phytase activity was evaluated measuring the production of *halo zones* around the microbial colonies on a Phytate Screening Medium (PSM: 1% D-glucose, 0.4% Na-phytate, 0.2% CaCl_2_, 0.5% NH_4_NO_3_, 0.05% KCl, 0.05% MgSO_4_ ∙ 7 H_2_O, 0.001% FeSO_4_ ∙ 7 H_2_O, 0.001% MnSO_4_ ∙ H_2_O, 1.5% agar, pH 7.0) as described in Palla et al*.*^27^. In particular, the isolates were grown overnight at 28 °C in 4 mL of PSM broth medium by shaking, and the microbial cultures were then spotted on the agar plate and incubated at 28 °C for 48 h. To eliminate false positive results, plates were counterstained using 2% (w/v) cobalt chloride and 6.25% (w/v) ammonium molybdate as described by Bae et al*.*^52^ and by Pepe et al*.*^53^. The phytase ability of isolates was calculated as the difference between the total diameter (colony + *halo zone*) and the colony diameter.

The antioxidant activity was determined by the free radical scavenging capacity using the stable 2,2-diphenyl-1-picrylhydrazyl radical (DPPH). Yeasts were grown at 25 °C in Wheat Flour Hydrolysate (WFH) by shaking for 24 h. The WFH was produced as described by Coda et al*.*^54^. Four mL of each microbial culture were centrifuged at 8,000 rpm for 10 min, and 75 µL of supernatant were transferred into a test tube containing 1 mL of distilled water and 25 µL of DPPH solution. WFH not inoculated with yeast culture was used as a control. Absorbance at 517 nm was measured after 30 min of incubation in the dark by a spectrophotometer (Varian Cary 1E UV–Vis, Palo alto, CA, USA). The free radical scavenging capacity was quantified as follows: DPPH scavenging activity (%) = [(blank absorbance − sample absorbance)/blank absorbance] × 100 and expressed as nmol TEAC/mL (Trolox equivalents of antioxidant capacity).

### Molecular characterization of selected isolates

Based on in vitro screening results, the best 55 performing yeasts were selected. Among them, 31 had been previously identified^19, 27^ and the remaining 24 were identified by the amplification of the D1/D2 domains of the 26S rRNA gene and sequencing. DNA of isolates was extracted from microbial liquid cultures grown at 25 °C in YEPD broth, using a “MasterPure Yeast DNA Purification Kit” (Epicentre) as described by Agnolucci et al*.*^55^. PCR reaction was carried out using NL1 (5′-GCA TAT CAA TAA GCG GAG GAA AAG-3′) and NL4 (5′-GGT CCG TGT TTC AAG ACG G-3′) primers^56^ in a final volume of 50 μL, containing 5 μL of 10X DyNAzyme Buffer (Finnzymes, Thermofisher, Milan, Italy), 0.2 mM of each dNTPs (BioLabs), 0.5 µM of each primer (Eurofins Genomics, Ebersberg, Germany), 0.625U of Taq DyNAzyme II DNA polymerase (Finnzymes, Thermofisher, Milan, Italy) and using 10–20 ng of DNA. The amplifications were carried out with an iCycler-iQ Multicolor Real-Time PCR Detection System (Bio-Rad) using the following conditions: 94 °C initial denaturation for 1 min; 35 amplification cycles of 30 s at 94 °C, 30 s at 58 °C, 30 s at 72 °C; final extension at 72 °C for 5 min. The presence of amplicons was confirmed by electrophoresis in 1.5% (*w/v*) agarose gel stained with 0.5 µg/mL of REALSAFE Nucleic Acid Staining (Real laboratory SL, Valencia, Spain). All gels were visualized by UV and captured as TIFF format files by the UVI 1D v. 16.11a program for FIRE READER V4 gel documentation systems (Uvitec Cambridge, Eppendorf). PCR products were purified with the UltraClean PCR CleanUp kit (MO BIO Laboratories, Carlsbad, CA, USA) according to the manufacturer’s protocol, quantified and 5′ sequenced by Eurofins Genomics (Ebersberg, Germany). Sequences were analyzed using BLAST on the NCBI web (https://blast.ncbi.nlm.nih.gov/Blast.cgi). The related sequences were collected and aligned using MUSCLE^57, 58^, and phylogenetic trees were constructed using the Neighbor-Joining method based on the kimura 2-parameter model^59^ in Mega X software (https://www.megasoftware.net/) with 1000 bootstrap replicates^60^. The sequences were submitted to the GenBank of National Center for Biotechnology Information (NCBI), under the accession numbers from MT269548 to MT269571.

According to the identification results, 39 yeasts were selected and molecularly characterized. The intraspecific diversity of such isolates, along with the two commercial baker’s yeasts, was carried out by inter-delta region analysis. Amplification reaction was performed using δ_1_ (5′-CAAAATTCACCTATA/TTCTCA-3′) and δ_2_ (5′-GTGGATTTTTATTCCAACA-3′)^61^ primers (Eurofins Genomics, Ebersberg Germany) and 160 ng of DNA, as reported in Palla et al*.*^19^. All gels were visualized and captured as previously described. Inter-delta profiles were digitally processed and analyzed with the BioNumerics software version 7.6 (Applied Maths, St-Martens-Latem, Belgium). Profiles were compared using the band matching tool with a position tolerance and optimization of 0.5%, and similarity was calculated using the Dice’s coefficient. For cluster analysis, unpaired group method with arithmetic average (UPGMA) trees with highest resampling support, in a permutation sample of size 200, were constructed. The reproducibility of fingerprints was assessed on four randomly selected strains.

### Leavening ability of selected yeast strains

The 39 selected yeasts were further analyzed for their leavening ability, an important pro-technological feature, by in vivo screening using the five different wholegrain flours previously described. To this aim, yeast strains were cultivated into YEPD broth at 25 °C overnight; cells were then harvested following centrifugation (10,000 × yeast strains were cultivated into YEPD broth at 25 °C overnight; cells were then harvested following centrifugation (10,000 × g, 10 min, 4 °C), washed in sterile saline-peptone water (0.9% NaCl, 0.1% bacteriological peptone, Oxoid, Milan, Italy), re-suspended in tap water at the cell density of ca. 6.0—7.0 Log cfu/mL and used as starters for dough fermentation. Three different batches of each flour (62.5 g) and tap water (26.5 mL), containing the above cellular suspension of each yeast (cell density in the dough of ca. 6.0 log cfu/g), were pooled and used to prepare 100 g of dough with a dough yield of 160 (dough yield = dough weight × 100/flour weight) supplemented with chloramphenicol (0.1 g/L). Mixing was done manually for 5 min. Doughs were fermented at 30 °C for 24 h, according to the common temperature used in sourdough preparation at artisanal and industrial levels^62^*.* Not inoculated doughs (control) and Zeus IBA and Lievitalia commercial baker’s yeasts were used as references. The values of dough pH, before and after fermentation, were determined by a pHmeter (Medidor PH BASIC 20, Crison, Milan, Italy) with a penetration probe. During fermentation, the increase in volume (ΔV, cm^3^*)* was manually monitored every two hours and the leavening performance calculated as the increase in volume during the time of fermentation (ΔV/t, cm^3^/h)^19, 63^.

### In vivo functional properties of selected strains

The ten best performing yeasts for each flour were selected and further analyzed for their functional traits, i.e. phytase activity and content of polyphenols, by in vivo screening.

Water/salt-soluble extracts (WSE) of fermented doughs were prepared according to Weiss et al.^64^*. P*hytase activity was assessed by monitoring the rate of hydrolysis of *p*-nitrophenyl phosphate (*p*-NPP) as described by Rizzello et al*.*^65^. The assay mixture contained 200 μL of 1.5 mM *p*-NPP (Sigma, 104-0) (final concentration) in 0.2 M Na-acetate, pH 5.2, and 400 μL of WSE diluted tenfold. The mixture was incubated at 45 °C for 10 min and the reaction was stopped by adding 600 μL of 0.1 M NaOH. The *p*-nitrophenol released was determined by measuring the absorbance at 405 nm. One unit (U) of activity was defined as the amount of enzyme required to liberate 1 μmol/min of *p*-nitrophenol under the assay conditions.

The analysis of the total phenol contents was carried out on the methanolic extracts (ME) of fermented doughs by monitoring the reaction between phenols and the Folin–Ciocalteu reagent. In order to obtain the ME, two and half grams of each dough were mixed with 15 ml of 80% methanol. The mixture was sonicated for 30 min, with a Bransonic 3510 ultrasonic apparatus (Branson, Danbury, CT, USA), and centrifuged at 10,000×g for 30 min at 4 °C. ME were transferred into test tubes and stored at − 20 °C before analysis. The concentration of total phenols was determined as described by Slinkard and Singleton^66^, with some modifications. The reaction mixture contained 50 μL of ME, 250 μL of Folin-Ciocalteu reagent (Sigma Chemical Co.) and 450 μL of distilled water. After 10 min of incubation at room temperature, 1.25 mL of a 20% sodium carbonate solution were added. The mixture was incubated at room temperature for 20 min and the absorbance at 735 nm was determined. The concentration of total phenols was calculated as gallic acid equivalents (GAE).

The combination of leavening ability, phytase activity and total phenol content allowed the detection of the two best performing strains for each flour. Such selected fermented doughs, along with the dough fermented by the commercial baker’s yeast Zeus IBA and control dough, were further analyzed for their antioxidant capacity, phenolic acid, xanthophyll and anthocyanin contents using the methods described above.

### Statistical analysis

Analyses were carried out in triplicate with three biological replicates for each condition. The SPSS version 23 software (IBM Corp., Armonk, NY, USA) was used for the statistical analysis of phenolic acids, total anthocyanins, xanthophylls and total antioxidant capacity in the stone-milled cereal flours and in the selected fermented doughs of each cultivar. SPSS was also used for the statistical analysis of the leavening ability, phytase activity and phenol content of doughs singly inoculated with the yeast strains. Data were subjected to one-way analysis of variance (ANOVA) followed by Tukey’s HSD test at a 99% confidence level to evaluate the differences among strain traits. When data did not fit ANOVA assumptions, the Welch robust test of equality of means was performed.

The normalized values of leavening ability, phytase activity and phenol content were subjected to permutation analysis using PermutMatrix ver. 1.9.4^67, 68^.

## Supplementary information

Supplementary information.

## Data Availability

The datasets analysed during the current study are available from the corresponding author on reasonable request.
