# Supplementary material for: Characterization and selection of functional yeast strains during sourdough fermentation of different cereal wholegrain flours
Source: Sci Rep. 2020 Jul 30;10:12856. doi: 10.1038/s41598-020-69774-6 (PMC7393511; doi:10.1038/s41598-020-69774-6)
Supplement: Supplementary file 1 — Supplementary information. [file 41598_2020_69774_MOESM1_ESM.docx]

**Characterization and selection of functional yeast strains during sourdough fermentation of different cereal wholegrain flours**

Michela Palla, Massimo Blandino, Arianna Grassi, Debora Giordano, Cristina Sgherri, Mike Frank Quartacci, Amedeo Reyneri, Monica Agnolucci, Manuela Giovannetti

**Supplementary Table S1.** Cell wall-bound phenolic acids (CWBPAs), soluble (free and conjugated forms) phenolic acids (SPAs), total anthocyanin (TAC), xanthophyll (lutein and zeaxanthin) content and antioxidant capacity (AC) detected in the stone-milled flours of the tested cereals.

|  | Common wheat | | | | | | Emmer | | Barley | |  |  |
| --- | --- | --- | --- | --- | --- | --- | --- | --- | --- | --- | --- | --- |
| Parameters | red | | yellow | | blue | |  |  |  |  |  |  |
|  | grained | | grained | | grained | | Giovanni | |  |  |  |  |
|  | Aubusson | | Bona vita | | Skorpion | | Paolo | | Rondo | | SEM | P(F) |
| CWBPAs^1^ (mg/kg) | 710 | b | 765 | b | 897 | a | 575 | c | 957 | a | 17 | <0.001 |
| Hydroxybenzoic acid (mg/kg) | 2.69 | c | 4.27 | b | 5.04 | a | 1.96 | d | 2.58 | c | 0.11 | <0.001 |
| Vanillic acid (mg/kg) | 4.53 | c | 5.36 | bc | 5.67 | ab | 3.43 | d | 6.34 | ab | 0.24 | <0.001 |
| Caffeic acid (mg/kg) | 5.93 | b | 5.4 | b | 6.31 | b | 3.91 | c | 32.5 | a | 0.31 | <0.001 |
| Syringic acid (mg/kg) | 2.33 | c | 4.38 | b | 5.13 | a | 1.96 | c | 1.7 | c | 0.31 | <0.001 |
| p-Coumaric acid (mg/kg) | 17.6 | d | 24 | c | 36.6 | a | 16.5 | d | 29.8 | b | 0.7 | <0.001 |
| Ferulic acid (mg/kg) | 646 | c | 672 | c | 792 | b | 515 | d | 862 | a | 15 | <0.001 |
| Sinapic acid (mg/kg) | 30.9 | b | 50.5 | a | 46.2 | a | 31.8 | b | 21.7 | c | 1.6 | <0.001 |
| SPAs^1^ (mg/kg) | 63.6 | bc | 62.1 | c | 84.7 | a | 77.8 | ab | 37.2 | d | 4 | <0.001 |
| Hydroxybenzoic acid (mg/kg) | 2.4 | b | 2.64 | b | 3.96 | a | 3.09 | b | 1.15 | c | 0.22 | <0.001 |
| Vanillic acid (mg/kg) | 4.68 | c | 5.67 | b | 7.33 | a | 4.62 | c | 3.08 | d | 0.24 | <0.001 |
| Syringic acid (mg/kg) | 2.02 | c | 4.17 | b | 6.76 | a | 1.77 | c | 1.2 | c | 0.36 | <0.001 |
| p-Coumaric acid (mg/kg) | 1.69 | ab | 1.21 | c | 2.08 | ab | 1.19 | c | 1.49 | bc | 0.11 | 0.001 |
| Ferulic acid (mg/kg) | 17.6 | a | 15.7 | a | 20.8 | a | 16.1 | a | 17.8 | a | 1.2 | 0.081 |
| Sinapic acid (mg/kg) | 34.7 | b | 32.2 | b | 43.4 | a | 50.7 | a | 8.58 | c | 2.01 | 0.029 |
| TAC (mg cya/kg) |  |  |  |  | 22.8 |  |  |  |  |  |  |  |
| Lutein (mg/kg) |  |  | 3.35 | a |  |  | 1.98 | b |  |  | 0.06 | <0.001 |
| Zeaxanthin (mg/kg) |  |  | 0.28 | a |  |  | 0.24 | b |  |  | 0.008 | 0.01 |
| AC-FRAP (mmol TE/kg) | 7.49 | bc | 6.41 | bc | 7.89 | b | 5.78 | c | 23.6 | a | 8.68 | <0.001 |
| AC-ABTS (mmol TE/kg) | 18.8 | b | 18.2 | b | 19.9 | b | 17.5 | b | 31.9 | a | 7 | 0.003 |

Data are expressed on a dw basis. Means followed by different letters are significantly different, according to the REGW-Q test (the ANOVA level of significance is shown in the table).

SEM, standard error of the mean

^1^ sum of the CWBPAs and the SPAs determined by means of the RP-HPLC

**Supplementary Table S2.** *In vitro* screening of phytase activity of 139 yeast isolates.

| Isolates | Halo zone (z) (mm) | Percentage of isolates (%) |
| --- | --- | --- |
| IMA BL11, BL12, BL14, BL16, BL19, GTS18Y | z ≥ 4 | 4 |
| *S. cerevisiae* IMA 36Y, 105Y, D1Y, D2Y, D4Y-D7Y, D9Y, D10Y-D12Y, D14Y, D17Y- D19Y, D23Y- D25Y, G1Y, L2Y, L10Y, L13Y-L15Y, L17Y- L25Y; *S. cerevisiae* ATCC 32167; IMA BL10, BL15, GT5Y, GT7Y, GTS1Y, GTS3Y, GTS6Y, GTS15Y, GTS19Y, GTS20Y, GTSXY, GTW1Y, GTW4Y, GTW14Y, M32Y, M72Y, S11D, W9D | 2 ≤ z < 4 | 36 |
| *S. cerevisiae* IMA 19Y, C11Y, D3Y, D8Y, D13Y, D15Y, D16Y, D20Y-D22Y, G2Y, G4Y, G9Y, L1Y, L3Y-L9Y, L11Y, L12Y, L16Y; *K. exigua* DBVPG 6956; IMA BL3, BL5, BL7, BL9, GTS2Y, GTS4Y, GTS5Y, GTS8Y-GTS10Y, GTS12Y-GTS14Y, GTS16Y, GTS17Y, GTS21Y, GTW2Y, GTW3Y, GTW6Y-GTW11Y, GTW15Y, M47Y | 0,42 ≤ z < 2 | 37 |
| *S. cerevisiae* IMA C1Y, C2Y, C13Y, C14Y, C16Y, C18Y, C20Y, C22Y, C24Y, G3Y, G6Y- G8Y, G10Y- G19Y; *K. humilis* DBVPG 7219, *K. humilis* DBVPG 6754; *K. humilis* IMA G23Y; IMA BL2, BL4, BL17, BL18; GTS11Y, GTW12Y, GTW13Y | n.d. | 23 |

n.d. non-detectable

**Supplementary Table S3.** Yeast isolates and reference strains used in this study.

| Strains^a^ | Source of isolation |
| --- | --- |
| *Saccharomyces cerevisiae* IMA C1Y, C2Y, C11Y, C13Y, C14Y, C16Y, C18Y, C20Y, C22Y, C24Y, D1Y-D25Y, G1Y-G4Y, G6Y-G19Y, L1Y-L25Y;  *Kazachstania humilis* IMA G23Y | Tuscan wheat sourdoughs [19] |
| *Saccharomyces cerevisiae* IMA 19Y, 36Y, 105Y | PDO Tuscan bread sourdough [27] |
| IMA GT5, GT7, GTS1Y-GTS6Y, GTS8Y-GTS21Y, GTSXY, GTW1Y-GTW4Y, GTW6Y-GTW15Y, M32Y, M47Y, M72Y, S11D, W9DY | Italian sourdoughs |
| IMA BL2-BL5, BL7, BL9-BL12, BL14-BL19 | Boza, a cereal-based fermented drink |
| *Saccharomyces cerevisiae* ATCC 32167 | Unknown |
| *Kazachstania humilis* DBVPG 7219^T^ | Bantu beer |
| *Kazachstania humilis* DBVPG 6754 | Sourdough, Finland |
| *Kazachstania exigua* DBVPG 6956 | Wheat sourdough, Italy |

^T^Type Strain.

^a^IMA=International Microbial Archives, Department of Agriculture, Food and Environment, University of Pisa, Pisa, Italy; ATCC=American Type culture Collection, Manassas, Virginia, USA; DBVPG=International Collection of Department of Agricultural, Food and Environmental Science, University of Perugia, Perugia, Italy.


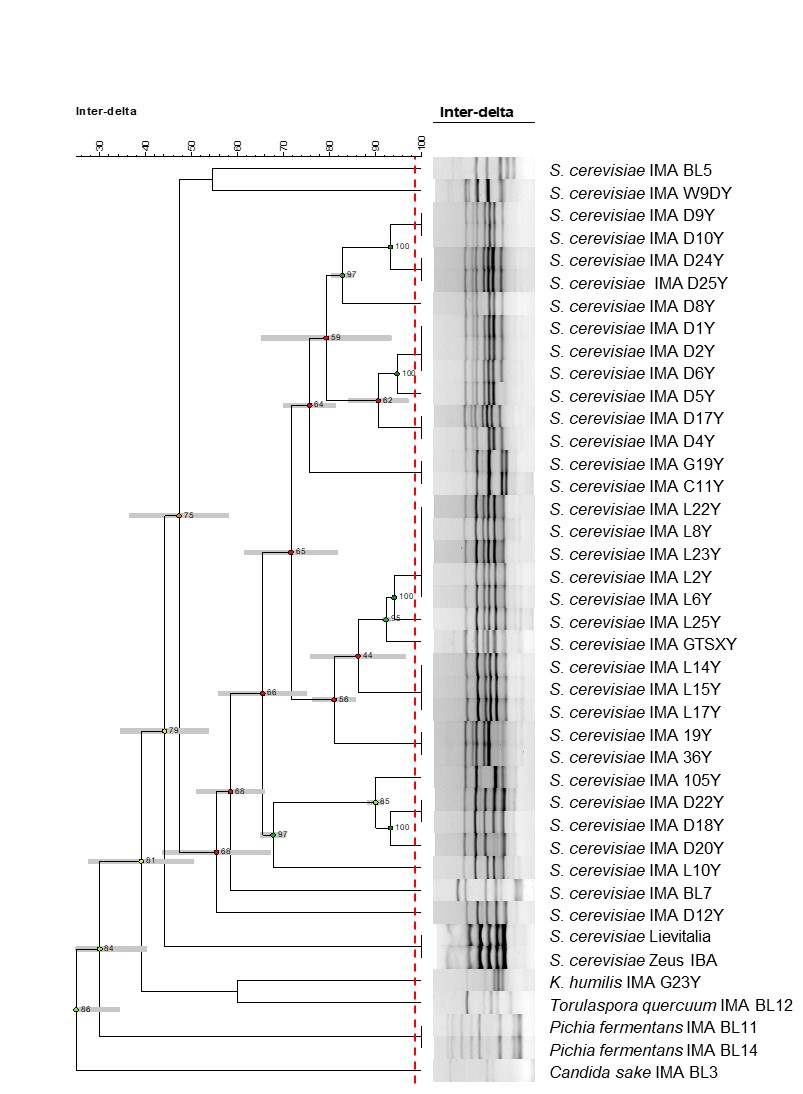


**Supplementary Fig. S1.** Dendrogram obtained from UPGMA analysis, using Dice’s coefficient, based on inter-delta profiles of the 39 selected yeast isolates and two commercial baker’s yeasts (Lievitalia and Zeus IBA). The red line indicates the similarity value (98.7%) for separation of biotypes. Standard deviation is shown at each node by a grey bar. Cophenetic correlation is shown at each branch by numbers and coloured dots, ranging between green-yellow-orange-red, according to decreasing values.
